# Trends in research on relationship between root exudates and soil phosphorus: a bibliometric analysis

**DOI:** 10.3389/fpls.2025.1616642

**Published:** 2025-07-31

**Authors:** Yijie Ding, Jiabao Yao, Fengqing Li, Shiyou Chen, Hui Wang, Lingyu Hou, Lei Liu, Qiwu Sun

**Affiliations:** ^1^ State Key Laboratory of Efficient Production of Forest Resources, Research Institute of Forestry, Chinese Academy of Forestry, Beijing, China; ^2^ Key Laboratory of Tree Breeding and Cultivation of State Forestry and Grassland Administration, Research Institute of Forestry, Chinese Academy of Forestry, Beijing, China; ^3^ Experimental Center of Subtropical Forestry, Chinese Academy of Forestry, Jiangxi, China

**Keywords:** root exudates, phosphorus, available nutrients, bibliometrics analysis, CiteSpace

## Abstract

**Introduction:**

Phosphorus (P) is an essential element for plant growth, but low availability of soil phosphorus limits agricultural and forestry development. Root exudates play a significant role in enhancing soil phosphorus availability.

**Methods:**

Based on bibliometric methods, this study conducted a quantitative analysis of 276 relevant articles from the Web of Science database between 2001 and 2024 using visualization tools such as CiteSpace and VOSviewer. The analysis explored multiple dimensions, including publication volume, contributing countries, highly cited literature, and keyword trends.

**Results:**

The results revealed that research in this field has gradually gained attention over the past two decades, with a rising trend in publications, particularly dominated by China. Studies primarily focused on botany, soil science, and agronomy, with frequent publications in journals such as Plant and Soil. Through clustering of high-frequency keywords related to root exudates and soil phosphorus interactions, research hotspots were found to have expanded from model plants and plant growth traits to diverse plant species and deeper mechanistic explorations.

**Conclusion:**

Future research should emphasize international collaboration, integrate controlled laboratory experiments with field trials, and leverage multidisciplinary approaches to advance practical applications, providing insights for addressing potential soil phosphorus crises and providing scientific support for the sustainable development of global agriculture and forestry and carbon neutrality goals.

## 1 Introduction

Phosphorus (P), a vital macronutrient, is involved in fundamental physiological processes (Akatsuki and Makita, 2020; McLaughlin et al., 2023), such as photosynthesis, enzyme regulation, energy metabolism and signal transduction in plants (Hinsinger, 2001; Bünemann, 2015). Plants primarily acquire and utilized P from soil in the form of inorganic Phosphorus (Pi) (Vance et al., 2003; Ceulemans et al., 2014). However, naturally acid soils occupy approximately 30% of the world’s ice-free land and are commonly associated with phosphorus (P) deficiency (Guo et al., 2010). The strong soil acidity in these regions promotes the binding of available P (primarily existing as orthophosphate ions) with iron and aluminum compounds (Bu et al., 2020; Yang et al., 2021), significantly reducing P bioavailability (Balemi and Negisho, 2012; Sharma et al., 2013). Consequently, P deficiency has become critical limiting factor for plant growth (Sun et al., 2021), threatening the sustainable development of agriculture and forestry. Although applying phosphate fertilizer, adjusting soil pH, and inoculating mycorrhizal fungi can effectively increase the available phosphorus levels in the soil (Gao et al., 2014; Zhan et al., 2015), excessive use of phosphate fertilizer may lead to environmental problems such as soil erosion and the depletion of non-renewable resources. Therefore, enhancing the phosphorus utilization efficiency of plants could be an effective approach in low - phosphorus environments.

Root exudates, comprising compounds either actively secreted or passively released by plant roots into the rhizosphere (Shen et al., 2005; Bhattacharyya et al., 2013; Wang and Liu, 2018), serve as mediators in the exchange of energy, matter, and information between plants and soil (Chen et al., 2002). Previous studies found that root exudates are essential for the activation of soil phosphorus and plant P acquisition efficiency (Vance et al., 2003; Liu et al., 2016). Specifically, plant roots can directly secrete acidic phosphatases or low-molecular-weight organic acids (e.g., citric, malic, and oxalic acids) to solubilize insoluble soil P compounds (Bertin et al., 2003; Cai Yinmei and Zhang, 2021), thereby enhancing phosphorus bioavailability for plant uptake. Therefore, investigating on the correlation between root exudates and soil phosphorus may represents an indispensable approach to alleviating the existing soil phosphorus crisis and upholding the stability of agro-forestry ecosystems. Current research on root exudates mainly focuses on collection methods, compositional profiles (Guo et al., 2019; Chen et al., 2023), spatiotemporal dynamics, and their roles in phosphorus activation (Yang et al., 2015, 2025). However, systematic studies on the organization, summarization, or quantitative analysis of root exudate-soil phosphorus relationships remain limited.

This study applies bibliometric methodologies to systematically analyze the interactions between root exudates and soil phosphorus. Using CiteSpace and VOSviewer, we conducted an in-depth bibliometric analysis of literature indexed in the Web of Science database from 2001 to 2024, aiming to: (1) Summarize the historical trajectory, thematic evolution, and key scientific outputs of root exudate-soil phosphorus research over the past two decades; (2) Characterize developmental patterns across multiple dimensions, including publication trends, contributions from key countries/regions, highly cited works, and emerging research frontiers; (3) Identify potential future directions for investigating the dynamic interplay between root exudates and soil phosphorus cycling.

## 2 Materials and methods

### 2.1 Data collection

Web of Science (WOS) is widely recognized as the authoritative and consistently curated database for high-impact scientific literature, standing as a key authoritative source of citation data (Chen et al., 2022). Considering its data standardization, a comprehensive literature review was performed utilizing the Web of Science Core Collection (WoSCC) database. The search strategy employed the following Boolean query, which is derived from the operators of the expanded database within the Web of Science’s Science Citation Index: TS = Topic; TI = Title; AND = Conjunction keyword; OR = Keyword group connection; * = Wildcard (for singular and plural suffixes). The search string is as follows: TOPIC = ((TS = root exudates*) AND (TI =“Phosphorus*”OR”phosphate*”)), covering the period from 1 January 2001 to 31 December 2024, with the search conducted on 10 January 2025, including English only. The data retrieved included a total of 527 papers. To refine the dataset and exclude irrelevant studies, duplicates, review, experimental methods, meeting, book, and research with non-relevant topics were filtered out, reducing the final dataset to 276 records. These publications from WoSCC were exported in plain text format.

### 2.2 Bibliometric analysis

**Figure 1:**
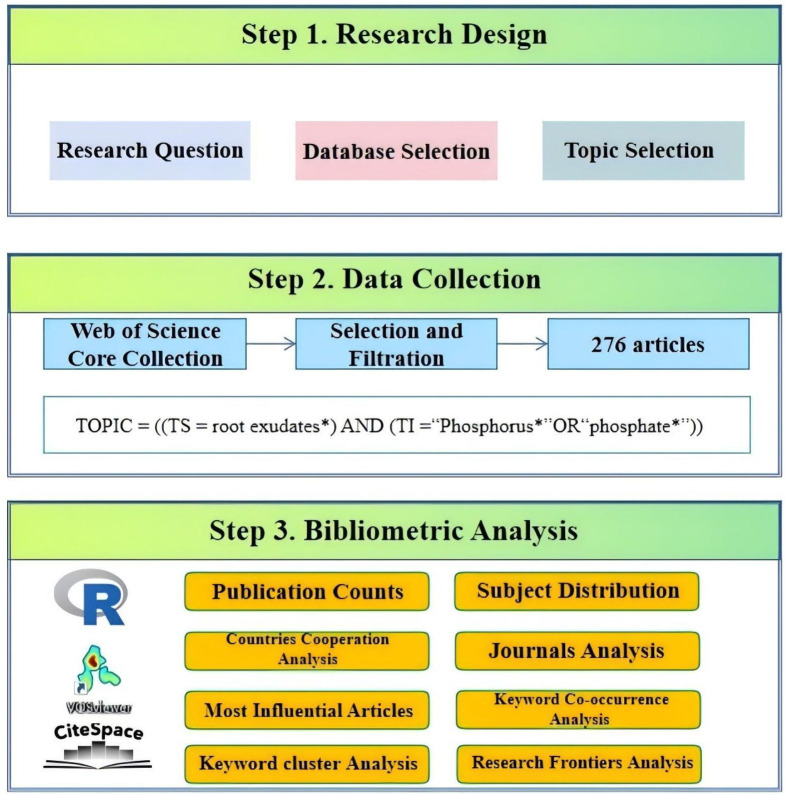
The flow chart of the literature search and bibliometric analysis, including three steps: (1) research design (2) data collection (3) bibliometric analysis.

The study adopted a systematic three-stage workflow to establish a comprehensive bibliometric analysis framework for this field (**Figure 1**).The first stage, the research design stage, identified the interrelationship between root exudates and soil phosphorus as the research theme and proposed three core research objectives. The second stage, the data collection phase, involved retrieving publications through advanced search queries in the target database. The third stage, the most crucial in the workflow, involved quantitative analysis of the 276 Web of Science articles published between 2001 and 2024 using CiteSpace (version 6.4.1) (http://citespace.podia.com, accessed on 10 January 2025), VOSviewer (version 1.6.20) (https://www.vosviewer.com, accessed on 10 January 2025) (van Eck and Waltman, 2010), and the R-based Bibliometrix package (version 4.3.1) (http://www.bibliometrix.org, accessed on 10 January 2025) (Aria and Cuccurullo, 2017). Publication counts, subject distribution, co-citation frequencies, international collaborations, and journal contributions related to root exudate-soil phosphorus interactions were analyzed using Microsoft Excel 2010 and the R-based Bibliometrix package, leveraging the bibliometric visualization and analysis functions of the Web of Science (WOS) database (https://bibliometric.com).

The Co-occurrence and Co-authorship networks in this field, including cooperative trends among countries, keyword Co-occurrence, and cluster analysis, were visualized using VOSviewer. Moreover, the top ten keywords with the most significant citation bursts were analyzed and mapped using CiteSpace. The time - zone map of research frontiers was drawn using CiteSpace, and the frontier trends in this field were identified through the method of keyword frequencies + burst term. The characteristics of the interrelationship between the root exudates and soil phosphorus field were systematically and objectively examined, with its research status, hotspots, and emerging trends delineated to facilitate a clearer understanding of its developmental trajectory and future prospects.

## 3 Results and analysis

### 3.1 Trend analysis of overall publications

**Figure 2:**
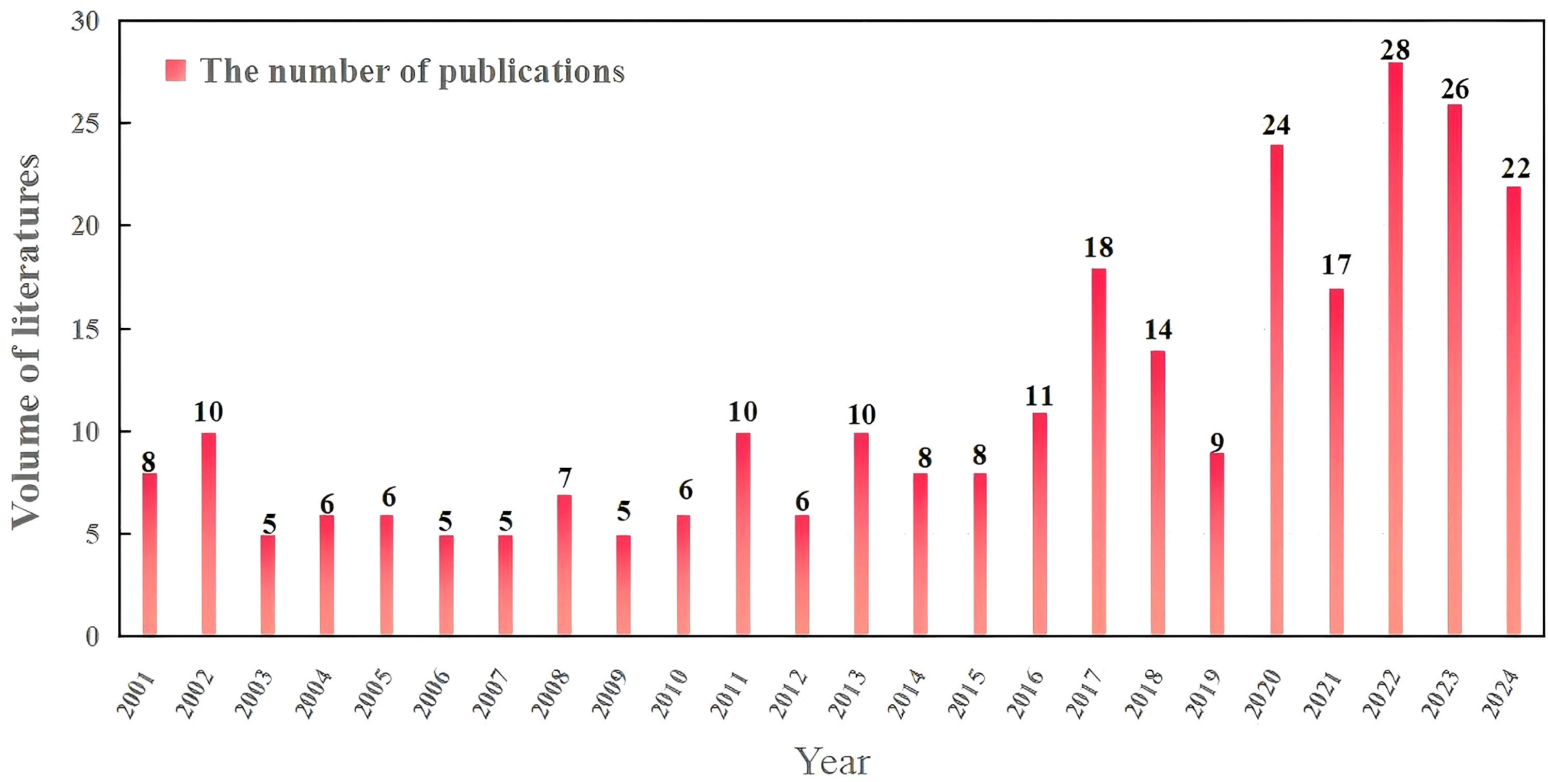
The number of publications in 2001 to 2024.

The annual trend of publications provides an intuitive measure of academic attention to research hotspots in specific fields (Tomasi et al., 2009). A bibliometric analysis of annual outputs from 2001 to 2024 reveals a consistent upward trend in studies on root exudate-soil phosphorus interactions (**Figure 2**). Based on publication patterns, the entire period was categorized into two distinct phases: an initial development phase (2001-2015) and a rapid growth phase (2016-2024).

During the initial development phase, annual publications remained below 10, though notable citation counts were observed in 2002, 2011, and 2013, suggesting influential early contributions. Since 2016, however, research activity has accelerated significantly, with annual outputs averaging 12 publications and exceeding 10 papers per year. In 2017, the annual publication count exceeded 15 for the first time, reaching a peak of 28 papers in 2022, reflecting a substantial surge in disciplinary interest and collaborative efforts.

From 2001 to 2015, research outputs in this field remained relatively limited, primarily attributed to the inherent complexity of the root exudate-soil phosphorus system and technological constraints of the era (Giles et al., 2012). The influence of root exudates on phosphorus transformation involves multidimensional factors, including soil minerals, microbial communities, and plant physiological regulation. The dynamic coupling mechanisms of these factors across spatial and temporal scales are highly complex, making related research more challenging. Soil type, climatic variability, and divergent agricultural management practices further limit the generalizability of research findings (Hocking and Jeffery, 2004; Plaxton and Tran, 2011). The collection and analysis of root exudates require high-precision equipment and meticulous operational protocols, posing significant challenges for conventional techniques to resolve their transient dynamics. The different types soil phosphorus (e.g., labile phosphorus, organic phosphorus, and stable phosphorus) require specialized techniques such as isotope labeling and Hedley phosphorus fractionation to study transformation mechanisms. Notably, around 2016, the research in this field experienced explosive growth, with the number of related papers reaching 28 by 2022, reflecting an impressive growth rate of 64.7% compared with the previous year. The occurrence of this phenomenon may be closely associated with multiple contributing factors. Firstly, advances in chromatographic techniques, such as high-performance liquid chromatography (HPLC) and gas chromatography-mass spectrometry (GC-MS) have enabled more precise compositional analysis of root exudates (Nourozi et al., 2024), allowing the identification of various substances including organic acids, sugars, and amino acids. When combined with metabolomic analysis methods, these techniques facilitate a deeper understanding of their roles in phosphorus transformation. Advances in soil nutrient analytical techniques, including X-ray diffraction (XRD) and phosphorus isotope tracing, has also provided more precise approaches for characterizing the content and spatial distribution of different phosphorus forms in soil (Ke et al., 2025). Furthermore, the development of high-throughput sequencing technology have facilitated a more comprehensive analysis of the structure and function of rhizosphere microbial communities, revealing the interaction mechanisms between root exudates and microorganisms (Vinothini et al., 2024). Similarly, the processes and functions of substance migration and transformation at the rhizosphere interface have been accurately captured and quantified due to the development and application of *in-situ* visualization techniques for the rhizosphere (Gu et al., 2024).

### 3.2 Analysis of research categories

**Figure 3:**
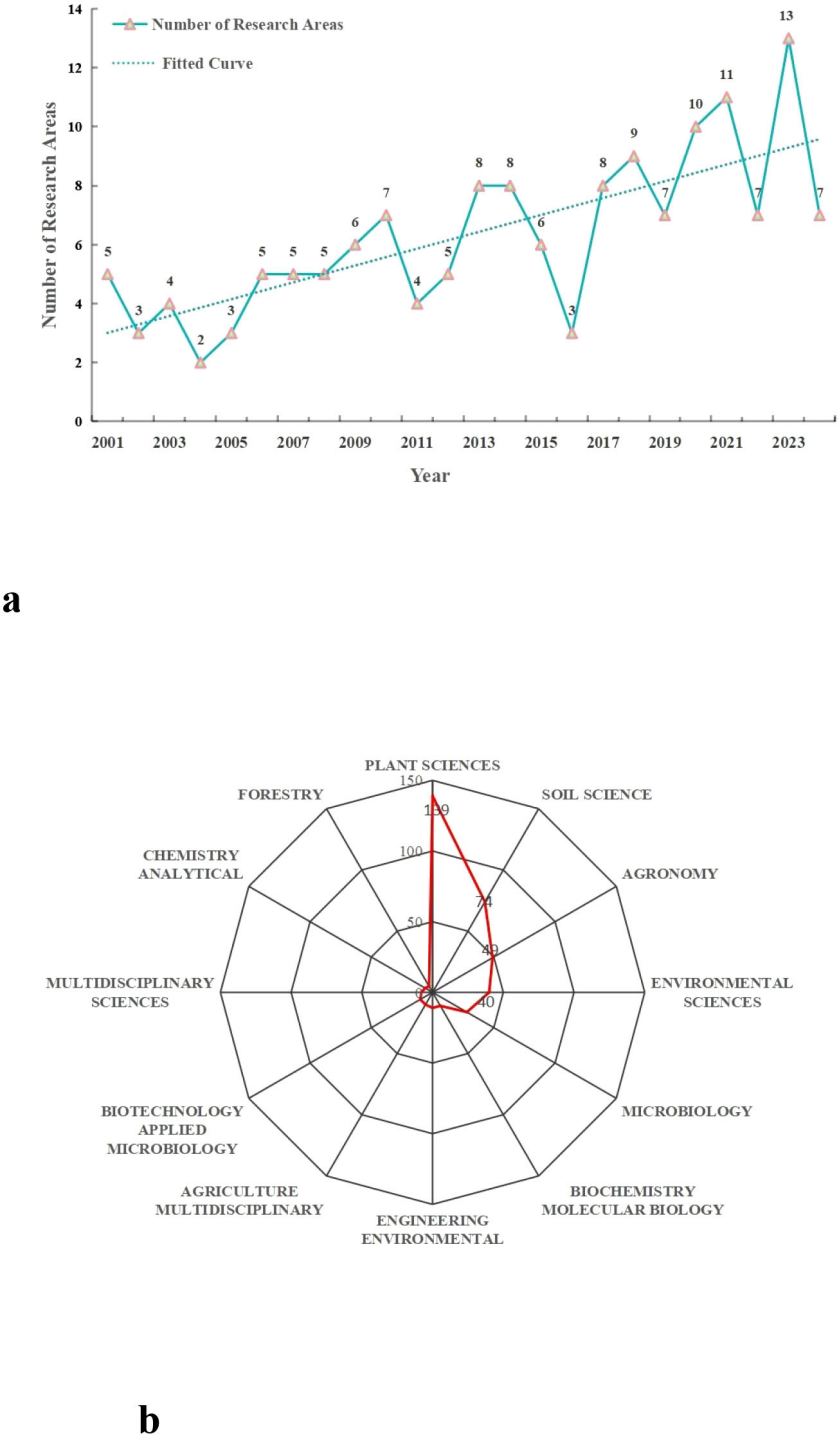
Temporal trends and categories distribution of WOS on the interrelationship between root exudates and soil phosphorus from 2001 to 2024. **(a)** Temporal trends in the number of WOS research Categories. **(b)** The top twelve categories distribution map.

This study reveals that research on the interaction between root exudates and soil phosphorus spans multiple categories. The number of these research categories covered by publications increased significantly from 5 in 2001 to 13 in 2023 (**Figure 3a**). This growth trend reflects the expansion of research into multidisciplinary domains as scientific investigations have deepened. From 2001 to 2024, the discipline with the highest publications was Plant Sciences (139 articles), followed by Soil Science (74 articles) and Agronomy (49 articles), which respectively accounted for 53.05%, 28.24%, and 18.70% of the total (**Figure 3b**). These findings indicate that the impact of root exudates on soil phosphorus transformation, particularly their effects on plants and crops, has remained a central focus of research in this field.

Additionally, the research categories also include Environmental Sciences, Microbiology, Biochemistry Molecular Biology, Engineering Environmental, Agriculture Multidisciplinary, Biotechnology Applied Microbiology, Multidisciplinary Sciences, Chemistry Analytical, Forestry. These categories provide valuable insights from diverse perspectives to further our understanding of the interactions between root exudates and soil phosphorus.

### 3.3 Analysis of active countries

**Figure 4:**
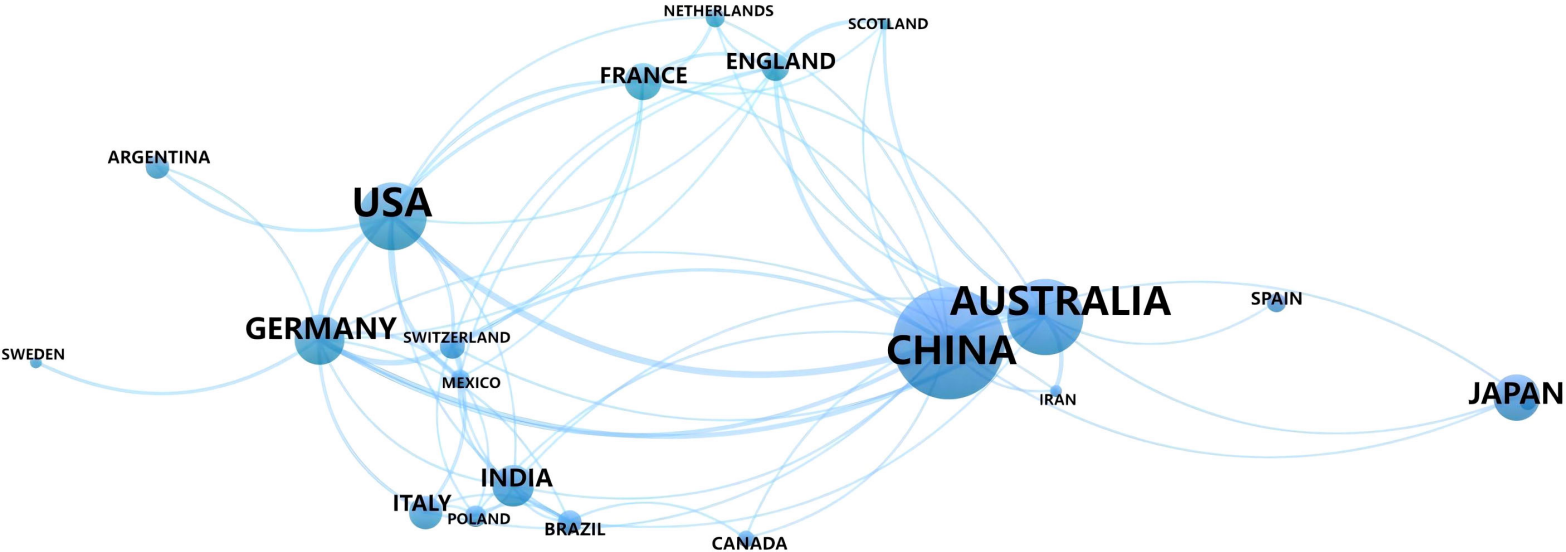
Cooperation between paper contributing countries.

Twenty countries have conducted research on the interactions between root exudates and soil phosphorus between 2001 and 2024. The top ten countries in terms of publication output account for approximately 94.93% of global research contributions in this field, with a total of 262 publications (**Table 1**). China has the highest publication output in this field (69 articles) (**Table 1**), which is likely closely associated with Chinese government policies that prioritize improving soil phosphorus availability and enhancing phosphorus fertilizer use efficiency. Additionally, researchers from 15 other countries conducted studies on the correlation between plant root exudates and soil phosphorus (**Table 1**; **Figure 4**). Similarly, China has the highest total citation frequency at 2,231 times (32 citations per article), suggesting a significant scholarly impact and a pronounced research advantage on the global stage. Although it has the highest publication output, its relatively lower citations per article indicate scope for enhancing research quality and depth.

**Table 1:** Number of country communications Top 10.

Rank	Country	Documents	Total link strength	Total citation	Average citation
1	CHINA	69	31	2231	32
2	AUSTRALIA	42	23	2208	53
3	USA	36	24	1767	49
4	GERMANY	24	26	1399	58
5	JAPAN	22	2	1019	46
6	INDIA	19	7	553	29
7	FRANCE	16	13	1041	65
8	ITALY	14	7	518	37
9	UNITED KINGDOM	11	12	1166	106
10	SWITZERLAND	10	17	662	66

Total link strength (Tls) serves as a critical metric for quantifying collaboration intensity between countries and regions. A higher Tls value indicates a stronger and more extensive collaborative network between the country or region and others (Wang and Tian, 2013). Among them, China (Tls=31), Australia (Tls=23), Germany (Tls=26), and the United States (Tls=24) rank highest, indicating that these countries are relatively active in research and collaboration within this field (**Table 1**). Further integrated analysis of the cooperation between countries using VOSviewer visualization software revealed that China and Australia have particularly close collaboration in this field (**Figure 4**), likely due to their shared research goals regarding the low efficiency of phosphorus utilization in soils (Cai et al., 2017). In the future, as global demand for sustainable agriculture and forestry increases, it is expected that cooperation between China and countries such as Australia, the United States, and Germany in areas like plant-microbe interactions and phosphorus use efficiency will further deepen.

### 3.4 Analysis of journal

Statistical analysis of journals with high overall publication output revealed that among the top 10 journals in terms of publication quantity, 29 articles were published in Plant and Soil followed by *Plant Science* (10 articles) (**Table 2**). This results indicates that *Plant and Soil* accepts the highest number of articles in the fields of root exudates and phosphorus transformation, while also reflecting a high level of scholarly recognition for the journal’s prominence and influence in these research field. As an internationally renowned journal, *Plant and Soil* focuses on the intersection of plant biology and soil science, aiming to uncover the ecological processes of plant-soil interactions. By integrating multidisciplinary perspectives, the journal plays a crucial role in promoting the integrated development of plant science and soil science. The consistently growing publications of research on root exudates and phosphorus transformation in *Plant and Soil* reflects both its significant academic value in interdisciplinary research and its relevance to addressing the global challenge of efficient phosphorus utilization.

**Table 2:** The Top ten journals in related fields from 2001 to 2024.

Journal	Pa	Tls	Tc	IF(2024)
*Plant and Soil*	29	35	1907	3.9
*Frontiers in Plant Science*	10	10	249	4.1
*New Phytologist*	8	19	986	8.3
*Journal of Plant Nutrition*	7	12	216	1.6
*Journal of Plant Nutrition and Soils Science*	7	9	475	2.5
*Biology and Fertility of Soils*	6	7	315	5.1
*Environmental and Experimental Botany*	6	18	301	4.5
*Soil Science and Plant Nutrition*	6	8	124	1.9
*Applied Soil Ecology*	5	3	111	4.8
*Environmental Science & Technology*	5	5	139	11.4

Pa (Published articles) represents the number of Published articles in this Journal; Tls (Total link strength) is an important metric for assessing the level of collaboration between Journals; Tc (Total citation) represents the number of citations within the analyzed dataset that reference articles published in the journal; IF (Impact factor).

Further analysis of the top 10 most highly cited articles in the research fields of root exudates and phosphorus transformation on the Web of Science (WOS) (**Table 3**) revealed that the article with the highest citation frequency is “Strigolactones are transported through the xylem and play a key role inshoot architectural response to phosphate deficiency in nonarbuscular mycorrhizal host *Arabidopsis*” by Kohlen et al., published in Plant Physiology. This study has been cited 351 times. The study found that, in response to phosphate deficiency, strigolactones played a central role in regulating the shoot architectural response of *Arabidopsis thaliana*, which is a plant that cannot form arbuscular mycorrhizal symbiosis. Moreover, this study developed a comprehensive analytical system for strigolactones based on high-performance liquid chromatography, column chromatography, and multiple reaction monitoring-liquid chromatography-tandem mass spectrometry. This system enabled systematic separation, high-sensitivity identification, and precise quantification of strigolactones in root exudates of *Arabidopsis thaliana*. The results not only provide a standardized analytical method for the isolation and identification of hormone-related substances in root exudates, but also offer important references for recent multi-omics studies on the composition of root exudates.

**Table 3:** The Top ten totally cited articles from 2001 to 2024.

Reference	Journal	Total citation	Year
(Kohlen et al., 2011)	*Plant Physiology*	351	2011
(López-Ráez et al., 2008)	*New Phytologist*	330	2008
(Hütsch et al., 2002)	*Journal of Plant* *Nutrition And Soil Science*	330	2002
(Xu et al., 2007)	*New Phytologist*	283	2007
(Yoneyama et al., 2007)	*Planta*	274	2007
(Umehara et al., 2010)	*Plant and Cell Physiology*	255	2010
(Veneklaas et al., 2003)	*Plant and Soil*	245	2003
(Yang et al., 2002)	*Planta*	196	2002
(Asghar et al., 2002)	*Biology and Fertility of Soils*	192	2002
(Marschner et al., 2002)	*Plant and Soil*	176	2002

### 3.5 Analysis of keywords and research hotspot

#### 3.5.1 Analysis of high frequency keyword

**Figure 5:**
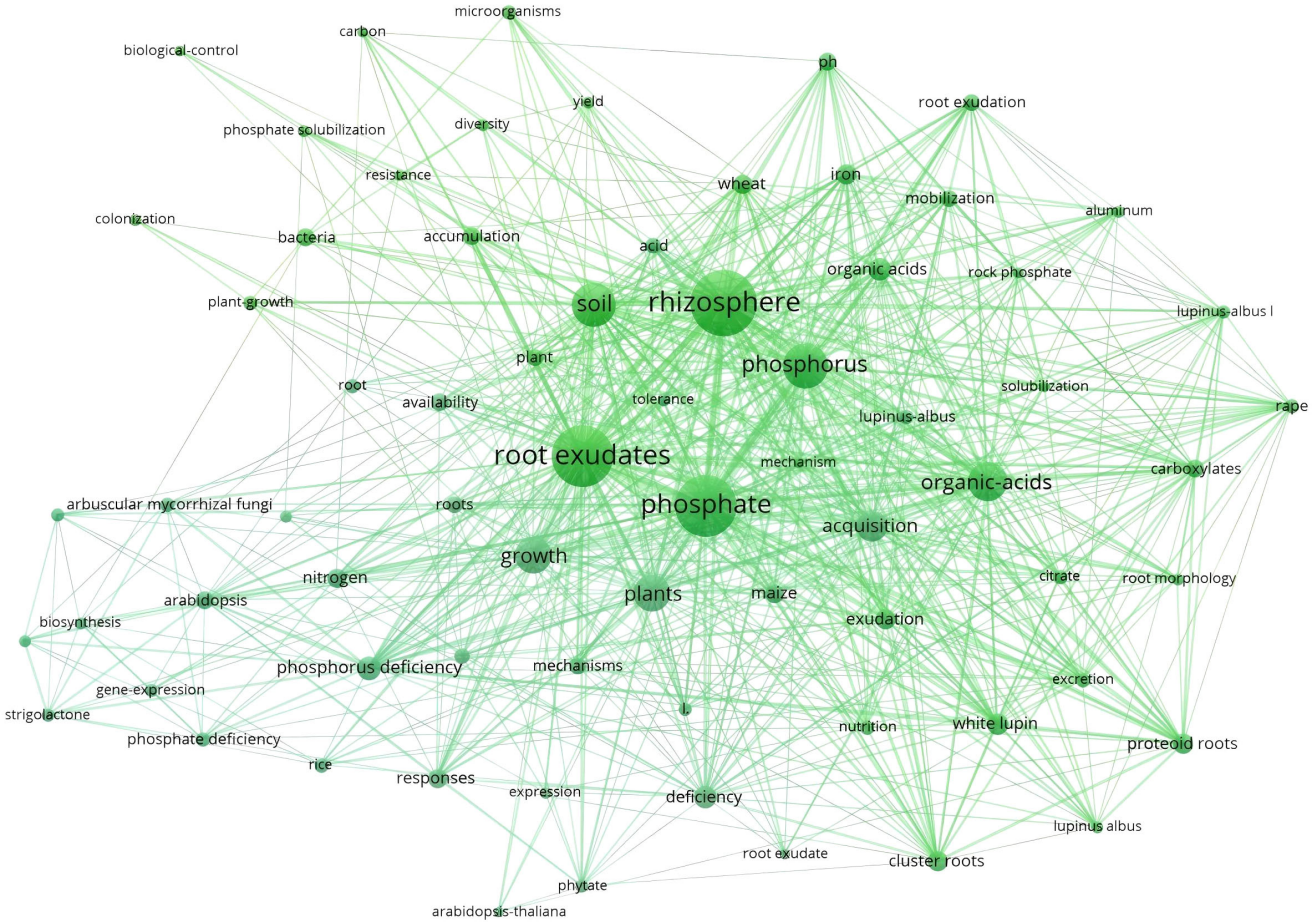
Keyword co-occurrence network in the WOS.

Keyword frequency analysis reveals the core themes and research hotspots (Chen et al., 2022). This study conducted a statistical analysis of the keywords in literature published in the WOS database between 2001 and 2024. A threshold of 8 occurrences was set for the literature on WOS, meaning only keywords that appeared at least 8 times were considered, and a total of 71 keywords were identified (**Figure 5**).

**Table 4** indicated the top twenty most frequent keywords in the dataset. Further analysis of keywords related to plant species-related revealed that the plants taxa with more than 10 occurrences in the dataset include “White lupin” (20 occurrences), “Wheat” (17 occurrences), “Maize” (17 occurrences), and “*Arabidopsis*” (15 occurrences). These results indicate that research on root exudates and phosphorus transformation predominantly focuses on agricultural plants, with relatively fewer studies on tree species relevant to forestry. The limited research on root exudates of forestry-related tree species restricts our understanding of key ecological processes within forest ecosystems, thereby hindering the sustainable development and full ecological functioning of forestry. Therefore, there remains significant potential for development in forestry-related research on the interrelationship between root exudates and soil phosphorus, requiring more attention and investment.

**Table 4:** Top 20 keyword of highly cited literature from 2001 to 2024.

Rank	Key words	Frequency	Rank	Key words	Frequency
1	Rhizophere	95	11	Deficiency	22
2	Root exudates	87	12	Organic acids	21
3	Phosphate	85	13	White lupin	20
4	Phosphorus	55	14	Proteoid roots	19
5	Soil	53	15	Iron	19
6	Organic-acids	46	16	Cluster roots	18
7	Plants	42	17	exudation	18
8	Growth	42	18	Wheat	17
9	Acquisition	32	19	Maize	17
10	P deficiency	23	20	Responses	17

#### 3.5.2 Analysis of keyword clustering

**Figure 6:**
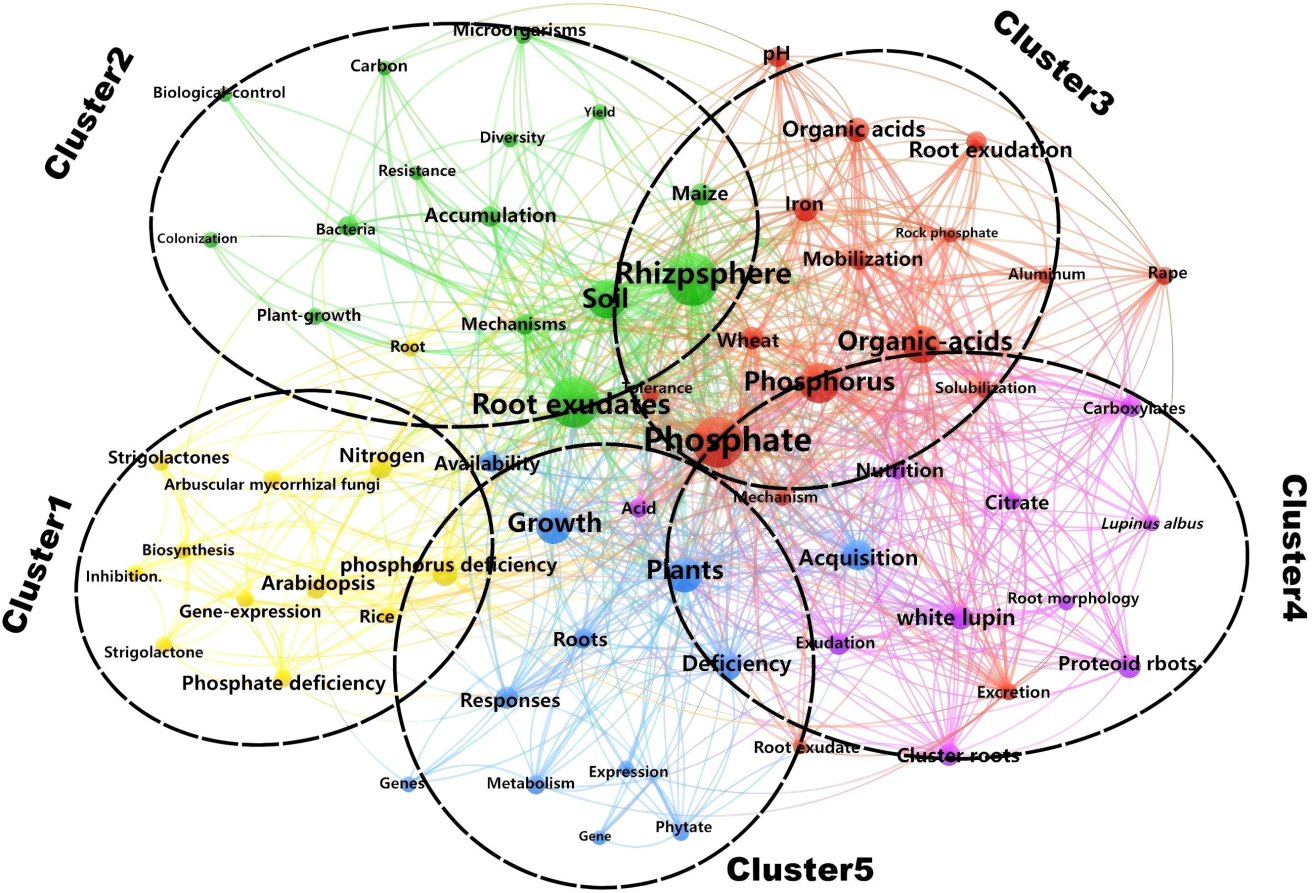
Keyword clustering network diagram in the WOS.

In this study, keyword clustering analysis was performed on 276 publications from the Web of Science (WOS) Database using CiteSpace software, with subsequent visual representation facilitated by VOSviewer software. The keywords were primarily grouped into five clusters (**Figure 6**).

The main keywords in Cluster 1 mainly include “phosphate deficiency”, “*Arabidopsis*”, “nitrogen”, and “gene expression”. The deficiency of available phosphorus in the soil prompts plants to activate adaptive mechanisms for insoluble phosphorus through root exudates, such as organic acids and phosphatases. This process involves changes in the composition of root exudates and their regulation of the rhizosphere microenvironment. Studies have found that differential expression of the *GASA* gene family in *Arabidopsis thaliana* under low phosphorus conditions may reveal its role in regulating root exudate synthesis or mycorrhizal symbiosis (Zhang and Wang, 2008). The introduction of nitrogen expands the dimension of nutrient interactions, and phosphorus stress may influence nitrogen uptake in plants by regulating the expression of nitrogen transport protein genes (such as the *NPF* gene family in *Arabidopsis*) (Wang et al., 2022). The keywords in Cluster 1 reflect a research continuum from “environmental stress” to “plant response” and further to “ecological interactions”, which highlights the interdisciplinary trends in researches on root exudates and phosphorus.

Cluster 2 includes the keywords “Root exudates”, “rhizosphere”, “Soil”, “microorganisms”, “maize”, and “mechanisms”. The rhizosphere serves as the pivotal hub of belowground plant-microbe interactions, governing the flow of matter, energy, and information within the plant-soil system (McLaughlin et al., 2023). Plants provide substrates and signaling molecules to rhizosphere microbes through root exudates, whereas microbes enhance soil phosphorus bioavailability through mineralization (Kaur et al., 2016; Akatsuki and Makita, 2020; Xu et al., 2025). Research have found that enhanced low molecular weight organic acid anions release in intercropping is beneficial for phosphorus acquisition of maize (Nong et al., 2022). Additionally, root exudates can also indirectly affect the functional diversity of microbial communities by altering the microecological structure of the rhizosphere, further enhancing the bioavailability of phosphorus (Vora et al., 2022). Cluster 2 focuses on the root exudate-microbe-soil interaction networks and their mechanisms in crops like maize, reflecting the central role of rhizosphere microecological processes driven by root exudates in phosphorus activation.

Cluster 3 includes the keywords “Phosphate”, “Organic-acids”, “Iron”, “Wheat”, and “mobilization”. In acidic or neutral soils, phosphorus often forms insoluble compounds with iron oxides (such as Fe^3+^), including iron phosphate (FePO_4_), which reduces phosphorus bioavailability (Jorgensen et al., 1996; Chen et al., 2005; Duan et al., 2020). Research has shown that plants significantly upregulate organic acid secretion in root exudates under phosphorus stress, activating insoluble phosphorus forms such as calcium phosphate (Ca-P), aluminum phosphate (Al-P), and iron phosphate (Fe-P) through acidification, complexation, or chelation (Sanuki et al., 2003; Kaur et al., 2016; An et al., 2022). As a pivotal global cereal crop, research on wheat’s phosphorus activation mechanisms of wheat generate novel strategies for agricultural and forestry production, whereby regulating organic acid secretion or breeding phosphorus-efficient cultivars, can both reduce reliance on phosphate fertilizers and enhance crop yields.

Cluster 4 focuses on the adaptive mechanisms of white lupin under phosphorus stress, and the keywords including “White lupin,” “Exudation”, “Cluster roots”, “Proteoid roots”, and “citrate”. As a typical model plant, white lupin forms a large number of proteoid roots (cluster roots) under phosphorus deficiency (Shen et al., 2005). This specialized structure expands the absorption surface area through dense arrangement, significantly improving the acquisition efficiency of insoluble phosphorus (Huyghe, 1997; Jeschke and Hartung, 2001). It also secretes high concentrations of citrate (Lambers et al., 2006), which not only acidifies the rhizosphere, chelates iron and aluminum ions, and disrupts phosphorus-metal oxide complex structures to activate insoluble phosphorus forms (e.g., Ca-P and Fe-P) (Ligaba et al., 2004), but also forms soluble complexes to promote root absorption (Yan et al., 2002). This cluster reveals the integrated mechanisms by which plants cope with phosphorus stress through dual strategies including morphological (proteoid roots) and biochemical adaptations (citrate secretion), thereby providing a framework for understanding plant-soil-microbe interactions.

Cluster 5 focuses on the systemic response mechanisms of plants to phosphorus deficiency, with keywords including “Plants”, “Growth”, “responses”, “acquisition”, and “deficiency” Upon encountering phosphorus deficiency, plants coordinate phosphorus acquisition through multi-level adaptive responses to maintain growth. This includes triggering root morphological remodeling (such as increased root length and root hair density) to expand the absorption surface area (Dinesh et al., 2022). Secondly, plants regulate soil phosphorus availability through root exudates (such as organic acids and phosphatases) (Tan et al., 2013). Whereas other clusters are primarily focused on a single model plant (e.g., *Arabidopsis thaliana*, rice and maize), this cluster emphasizes the characterization of conserved molecular pathways operative across diverse plant lineages. The study demonstrates that plants integrate physiological (e.g., root morphological changes), biochemical (e.g., exudate regulation), and molecular (e.g., gene expression) strategies to facilitate efficient phosphorus acquisition. Future research may further integrate single-cell transcriptomics to analyze the differential response among distinct root cell types, or employ high-throughput phenomics to identify phosphorus-efficient genotypes, thereby providing theoretical support for precise management of phosphorus fertilizer in sustainable agriculture and forestry.

### 3.6 Analysis of frontiers and future directions

**Figure 7:**
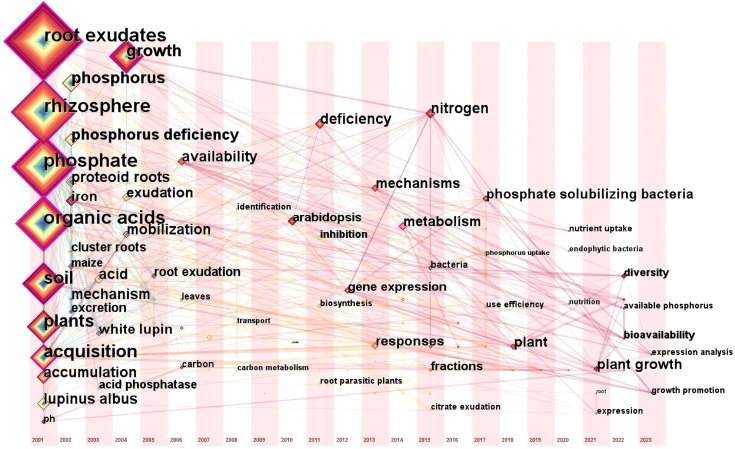
The view of frontiers in the WOS publication.

To further reveal the core research directions and evolving trends in this field, this study employed CiteSpace software to perform a temporal zone analysis of keywords from all relevant publications (2001-2024) in the Web of Science (WOS) database, and generated a visualization of frontier research frontiers (**Figure 7**). These results may enable a more precise analysis of the central themes in past and current research and, to some extent, predict future developments in the field. In the temporal visualization of research frontiers, the size of the node denoting a keyword is proportional to its appearance frequency; the density and length of linkages between nodes reflect the temporal span of that keyword. Analysis of the WOS research frontier temporal zone diagram reveal that keywords such as “Root exudates”, “Phosphorus”, “Organic acids”, “Soil”, “Plant”, and “*Lupinus albus*” exhibit the highest frequently and strongest connections to other keywords. These keywords have consistently emerged throughout the 2001–2024 period, indicating their significant role in the research on root exudates and phosphorus transformation.

**Figure 8:**
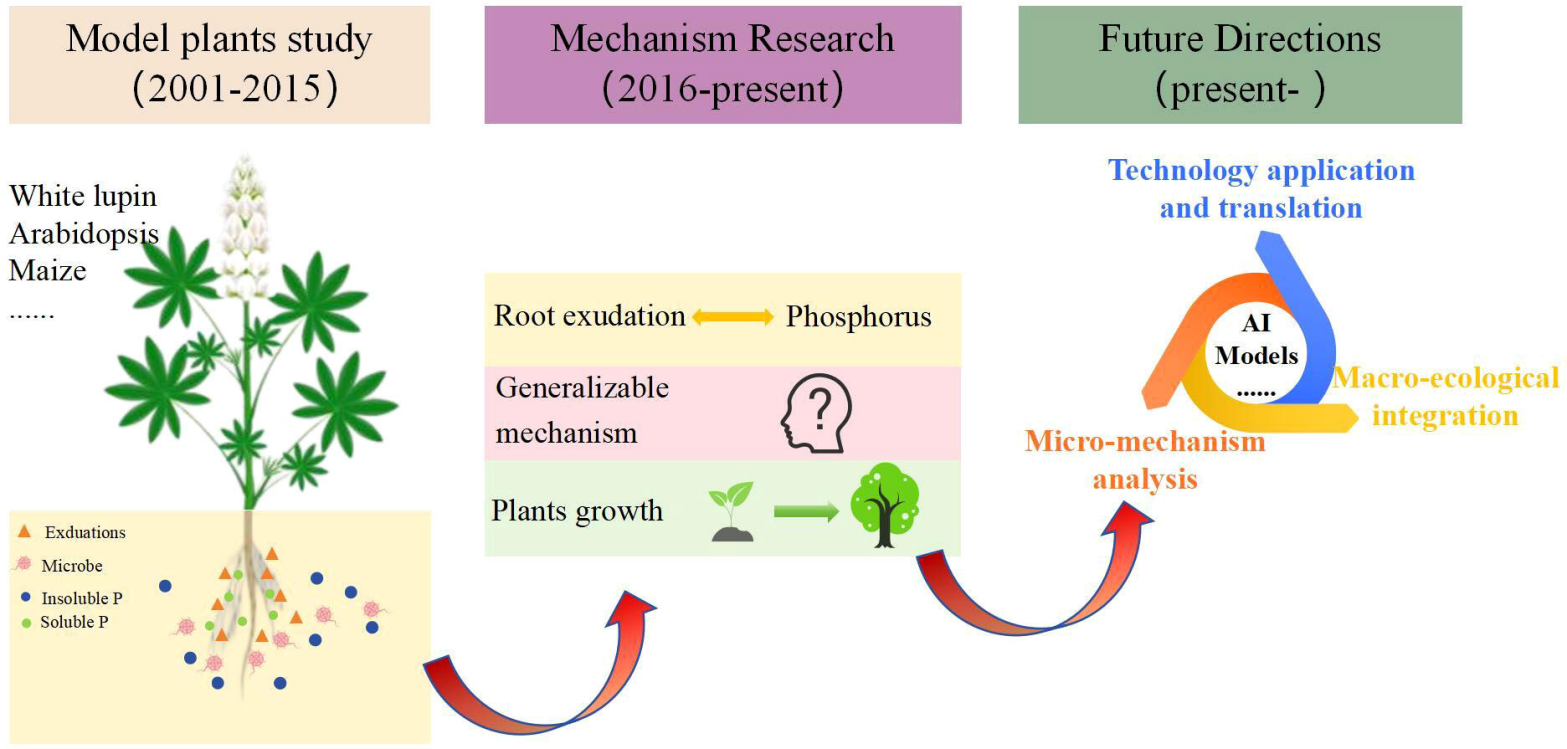
The development and perspectives of the interactions between root exudates and soil phosphorus.

To identify keywords that exhibited sudden citation bursts in specific years and uncover deeper disciplinary developments and emerging research frontiers, this study utilized the “Timezone” function in CiteSpace to perform burst analysis on keywords from the WOS database. The top 10 keywords with the strongest burst strength are listed in **Table 5**. An analysis of the burst timings for these keywords revealed that early researches focused on plant adaptive strategies, particularly the impact of root structure and exudates on phosphorus activation. Thus, “*Lupinus albus* L.” and “Proteoid roots” had the highest burst strength during 2002-2006. With the advancement of research techniques, the core theoretical frameworks underlying phosphorus activation mechanisms have been formulated. Meanwhile, research scope has expanded to encompass diverse plants species, as well as plant growth regulation, the synergistic effects of multi-nutrient interactions, thereby resulting in heightened burst strength for keywords such as “Plant,” “Growth,” and “Nitrogen” since 2018. These also reflect the dynamic interplay between academic advancements and societal demands. Future research should integrate microbial functional genes and microbial metabolic networks to further elucidate the mechanisms by which plant root exudates modulate rhizosphere microbial communities to promote soil phosphorus activation. Furthermore, gene editing and epigenetic regulation techniques should be utilized to improve plant adaptation to low-phosphorus soils environments. Research on root exudate-soil phosphorus interactions will advance towards an integrated framework that incorporates “micro-mechanism analysis—macro-ecological integration—technology application and translation”. Research hotspots will gradually shift toward four main directions: plant-microbe interactions, multi-nutrient coupling, gene editing, and smart agriculture and forestry (**Figure 8**). Furthermore, while the keyword “intercropping” did not rank among the top ten terms, it is undeniable that significant advancements have been made in research concerning soil phosphorus availability and root exudates within crop planting systems, rotations, and intercropping practices. These studies are transforming the agricultural landscape across Europe, the United States, and other countries. For instance, in the intercropping system of leguminous plants and cereal crops, the root system of leguminous plants releases more protons and organic acids, locally acidifying the rhizosphere soil and increasing the availability of phosphorus, thereby promoting the absorption of phosphorus by adjacent cereal crops (Yu et al., 2021). These research accomplishments also offer valuable insights for agricultural transformation in developing countries. Overall, investigations into soil phosphorus availability and root exudates are steering global agriculture and forestry toward a more sustainable future. This represents not only a scientific and technological advancement but also a crucial step for humanity in addressing forthcoming food security challenges.

**Table 5:** Information of Keywords with the citation burst from 2001 to 2024.

Keywords	Strength	Begin	End	Time (2001-2024)
Exudates	3.57	2002	2005	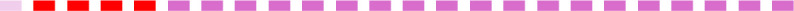
*Lupinus albus* L.	3.48	2002	2006	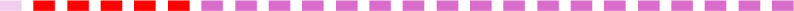
Proteoid roots	3.19	2002	2006	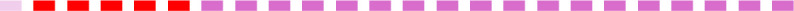
Phosphorus	2.93	2002	2010	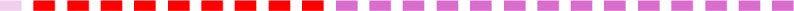
P. deficiency	2.71	2009	2015	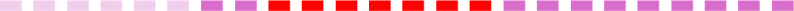
Lupinus albus	2.65	2009	2013	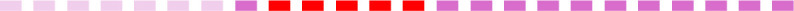
Responses	3.47	2017	2020	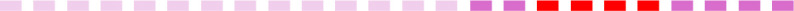
Plant	2.79	2018	2024	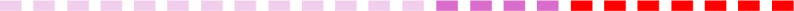
Growth	3.23	2019	2024	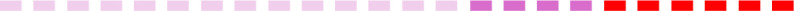
Nitrogen	3.21	2020	2024	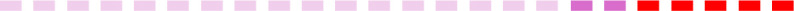

In the graphical representation, the purple lines indicate the time intervals and the red segments represent the periods of reference bursts.

### 3.7 Analysis of limitations

To better understand the interactions between root exudates and soil phosphorus, we counted 276 articles refined on WOS and systematically analyzed the overall trend of publications, research categories, active countries, journal, high frequency keyword, keyword Clustering, frontiers and future directions, revealing the core hotspots and developmental pathways in this field.

However, this study has several limitations that should be explained. First, this research exclusively utilizes articles sourced from the WOS database, it does not include publications from other databases such as Scopus or Google Scholar. The reason for this is that WOS is widely recognized as an authoritative and continuously maintained database for high-impact scientific literature, especially in the field of natural sciences. Additionally, bibliometric tools like CiteSpace and VOSviewer have been optimized to handle WOS data formats, and this compatibility minimizes technical errors during the construction of networks and cluster analysis. Finally, when integrating records from multiple databases, issues such as data deduplication, inconsistent domain coordination, and differences in term mapping are inevitable, which may compromise the visualization effect and the repeatability and validity of the indicators. To address this limitation, future studies could expand their scope by conducting multi-database bibliometric analyses, thereby ensuring a more comprehensive and representative assessment of the publications. Second, bibliometric methodologies predominantly rely on citation-based metrics, which may not serve as definitive indicators of scientific impact. Consequently, emerging research areas with relatively low citation counts may be underrepresented, despite their potential significance. Third, since WOS mainly indexes English-language publications, it could lead to the underrepresentation of significant works published in other languages. Future research should integrate multilingual datasets to improve the inclusivity and comprehensiveness of bibliometric evaluations.

## 4 Conclusion

This study focuses on the interactions between plant root exudates and soil phosphorus using 276 relevant articles published between 2001 and 2024 in the WOS database. Bibliometric analysis was systematically analyzed the research landscape, revealing the core hotspots and developmental pathways by examining publication trends, global distribution, highly cited papers, and emerging themes. The findings provide a data-driven foundation for optimizing phosphorus use efficiency via root exudate mechanisms in agricultural and forestry ecosystems.

(1) Due to the complexity of the research system on the relationship between root exudate and soil phosphorus and the limitations of technology, the overall number of relevant publications is relatively small. However, from 2001 to 2024, the number of publications showed a general upward trend, peaking in 2022. In terms of countries with publications, China ranks first with 69 papers, but the average citation frequency per paper is relatively lower. In the future, it is necessary to strengthen international cooperation and enhance the innovation of basic research. Regarding the disciplinary classification, the research on the relationship between root exudates and soil phosphorus is widely distributed across a wide range of fields, yet the forestry research is relatively scarce. Similarly, the frequency of key keywords indicates that current studies mainly focus on agricultural crops, with few studies on forestry related tree species. Future research should prioritize investigations into key forestry tree species to enhance phosphorus use efficiency and drive sustainable development in forest ecosystems.

(2) Clustering analysis grouped the keywords into five clusters. Clusters 1 to 4 focus on *Arabidopsis*, maize, wheat, and white lupin, respectively, highlighting key hotspots and challenges such as gene expression regulation, root exudate-microbe-soil interaction networks, and the transformation mechanisms of insoluble phosphorus. Notably, Cluster 5 transcends single model plants by revealing systemic responses and generalizable mechanisms under phosphorus deficiency stress, exemplifying the shift from species-specific to pan-plant research frameworks.

(3) The study of the relationship between root exudates and soil phosphorus is an interdisciplinary field. Future research should deeply integrate gene regulation, functional microbiology, chemical structure analysis, and data-driven model prediction, thereby achieving a leap from “mechanism analysis of phosphorus activation” to “intelligent design of rhizosphere environments”. Concurrently, efforts are needed to explore pathways for translating research outcomes into practical applications by leveraging the inherent advantages of plants. By applying root exudate technology, this research can address the current deficiency of available phosphorus in soils, providing scientific foundations for global sustainable agriculture and forestry development and carbon neutrality goals.

## Data Availability

The original contributions presented in the study are included in the article/supplementary material. Further inquiries can be directed to the corresponding authors.
